# Photo-Oxidation Products of Skin Surface Squalene Mediate Metabolic and Inflammatory Responses to Solar UV in Human Keratinocytes

**DOI:** 10.1371/journal.pone.0044472

**Published:** 2012-08-30

**Authors:** Vladimir Kostyuk, Alla Potapovich, Andrea Stancato, Chiara De Luca, Daniela Lulli, Saveria Pastore, Liudmila Korkina

**Affiliations:** 1 Laboratory of Tissue Engineering & Skin Pathophysiology, Istituto Dermopatico dell'Immacolata (IDI IRCCS), Rome, Italy; 2 Biology Department, Belarus State University, Minsk, Belarus; North Carolina State University, United States of America

## Abstract

The study aimed to identify endogenous lipid mediators of metabolic and inflammatory responses of human keratinocytes to solar UV irradiation. Physiologically relevant doses of solar simulated UVA+UVB were applied to human skin surface lipids (SSL) or to primary cultures of normal human epidermal keratinocytes (NHEK). The decay of photo-sensitive lipid-soluble components, alpha-tocopherol, squalene (Sq), and cholesterol in SSL was analysed and products of squalene photo-oxidation (SqPx) were quantitatively isolated from irradiated SSL. When administered directly to NHEK, low-dose solar UVA+UVB induced time-dependent inflammatory and metabolic responses. To mimic UVA+UVB action, NHEK were exposed to intact or photo-oxidised SSL, Sq or SqPx, 4-hydroxy-2-nonenal (4-HNE), and the product of tryptophan photo-oxidation 6-formylindolo[3,2-b]carbazole (FICZ). FICZ activated exclusively metabolic responses characteristic for UV, i.e. the aryl hydrocarbon receptor (AhR) machinery and downstream *CYP1A1/CYP1B1* gene expression, while 4-HNE slightly stimulated inflammatory UV markers *IL-6*, *COX-2*, and *iNOS* genes. On contrast, SqPx induced the majority of metabolic and inflammatory responses characteristic for UVA+UVB, acting *via* AhR, EGFR, and G-protein-coupled arachidonic acid receptor (G2A).

**Conclusions/Significance:**

Our findings indicate that Sq could be a primary sensor of solar UV irradiation in human SSL, and products of its photo-oxidation mediate/induce metabolic and inflammatory responses of keratinocytes to UVA+UVB, which could be relevant for skin inflammation in the sun-exposed oily skin.

## Introduction

Numerous mechanisms have been evolved in human skin to sense environmental stimuli and to mount adaptive responses in order to maintain homeostasis in the skin and protect the entire organism. The ultraviolet component of solar light consists of UVB and UVA parts, which differentially penetrate the skin barrier and thus affect prevalently epidermal (UVB) or dermal (UVA) skin cells and corresponding extracellular structures/molecules [1]. Skin surface lipids (SSL) play an important role for essential human skin functions such as mechanical and chemical barrier, thermoregulation, and photo-protection [1]–[3]. Laying on the surface of the skin, SSL are also exposed to the highest doses of UVA+UVB and they form the first-line defence against its potential danger. Major photo-protective components of SSL are α-tocopherol and squalene (2,6,10,15,19,23,-hexamethyl-2,6,10,14,18,22-tetracosahexaene, Sq), both working as sacrificing antioxidants, since they block photo-induced lipid peroxidation in cellular and acellular skin components, either by chain-breaking mechanism (α-tocopherol) [1], [4], [5] or by quenching singlet oxygen (Sq) [6]. Both are continuously produced by skin surface-open sebaceous glands to maintain their physiologically essential levels and substitute photo-destructed molecules [1]–[3]. Sq is the most abundant oxidisable component of SSL, its concentrations in adult human skin reaching up to 20%, while its levels are negligible in other organs [2], [7]. Upon action of environmental oxidants and microbial residents of the skin, fast oxidative degradation of Sq occurs giving rise to a wide spectrum of by-products, such as monohydroperoxides, epoxides, and aldehydes (Fig. 7A) [1], [2], [8]. Physiological doses of UVA oxidise Sq at much higher rates than UVB [1], [2]. The role of Sq and its oxidation products (SqPx) in skin photo-protection [9] and in the induction of inflammatory responses of keratinocytes in the context of acne pathogenesis [10] has been evaluated and recently reviewed in [2], [3].

The search of endogenous extracellular sensors of UV mediating its effects on skin cells has received growing interest in the last years. Products of tryptophan photo-oxidation, 6-formylindolo[3,2-b]carbazole (FICZ) in particular, were extensively studied and shown to mimic UVB in the induction of aryl hydrocarbon receptor (AhR)-controlled metabolic cascade in hepatocytes and HaCaT [11]–[13] , and of melanogenesis in melanocytes [14]. AhR is a cytosol-associated and ligand-activated receptor with transcription factor functions. Upon stimulation by a ligand, AhR liberates from its chaperon heat shock protein 90 (Hsp90), co-chaperon p23, and XAP-2 and moves to the nucleus, where it binds to its specific nuclear co-partner Arnt to acquire full binding capacity to the promoter of target genes, first of all *CYP1A1* and *CYP1B1*, to start the metabolic program [12], [13]. Over-expression of CYP1A1 and CYP1B1 enzymes has been detected in UVB exposed human skin [15]. As shown recently, the physiological role of AhR is not limited to the control of xenobiotic metabolism, but it extends to numerous cell functions such as breakdown of endogenous metabolites, proliferation, cell-to-cell contacts, immune and inflammatory responses, melanogenesis, and circadian rhythm [16], [17]. Among the endogenous AhR ligands known so far [18], [19], there are the products of tryptophan photo-oxidation [13], prostaglandins [20], bilirubin/biliverdin [21], and kinurenic acid [22], all of them present in the skin cells/cellular membranes.

Another UV-sensitive receptor is the epidermal growth factor receptor (EGFR), located on the cellular membrane of keratinocytes, which is a key regulator of numerous essential processes underlying skin development, homeostasis, stress responses, and repair [23]–[26]. 4-Hydroxy-2-nonenal (4-HNE), the final product of membrane-bound arachidonic acid photo-oxidation, appears to be a signalling molecule of UVB-induced EGFR nuclear translocation in keratinocytes [27]–[29], where the receptor physically or functionally interacts with other transcription factors with DNA-binding activity, including the signal transducer and activator of transcription 3 (*STAT3*). These events lead to the up-regulation of distinct genes controlling cell proliferation, DNA repair, as well as *COX-2* and *iNOS* [23]. Moreover, the cytoplasmic components of the EGFR system, the EGFR-ERK1/2 or EGFR-phosphoinositol-3-phosphate (PI3P)-protein kinase B (Akt1) pathways, are involved in the regulated expression of inflammatory chemokines in normal human keratinocytes [23], [25], [30], including CXCL8/IL-8, a chemokine sensitive to UV irradiation and reasonably involved in UV-induced granulocyte recruitment to the irradiated skin sites as well as in solar hyperkeratosis [25].

Upon skin exposure to UVB, both keratinocyte membranes and intercellular epidermal lipids containing linoleic acid are oxidised and 9-hydroxyoctadecadienoic acid (9-HODA) is formed. 9-HODA is a ligand for G-protein coupled arachidonic acid receptor (G2A) on the keratinocyte membrane [31]. G2A is a stress-inducible receptor for lisophosphatidylcholine and sphingosylphosphorylcholine [32] and for oxidized free fatty acids [31]. The 9-HODA-dependent activation of G2A leads to over-expression of pro-inflammatory cytokines-markers for UVB (IL-6, IL-8, and IL-1) [31], [33].

The major goal of the present study was the search for a first-line endogenous extracellular UV sensor(s) located in the outmost skin surface lipids (SSL). Here, we evaluated the role of Sq, a major photo-oxidisable component of SSL, in sensing solar UVA+UVB and transmitting their signal to underlying skin keratinocytes. We showed for the first time that photo-oxidised SqPx directly applied to primary human epidermal keratinocytes (NHEK) mimics the majority of UVA+UVB effects towards primary human epidermal keratinocytes (NHEK), by stimulating the AhR-controlled metabolic pathway and affecting EGFR- and G2A-connected inflammatory responses.

## Materials and Methods

### Ethics Statement

All experiments with human material (skin biopsies and skin surface lipids) were carried out in accord with Helsinki Declaration, the protocols were approved by Ethical Committee of Istituto Dermopatico dell'Immacolata, Rome, and the healthy adult donors signed the informed consent.

### Skin surface lipid collection and analysis

SSL were obtained from sebum of 10 healthy adult male volunteers by non-invasive cup extraction method, by applying 10 ml diethyl ether on the upper dorsal skin with a glass cylinder with open ends (5.3 cm^2^), allowing contact for 2 min. Then, the freshly collected SSL extracts were pooled, filtered, and evaporated under nitrogen flux [7]. SSL collection was performed during autumn-winter period to avoid effects of recent sun exposure. The SSL were derivatized at 80°C for 30 min by the addition of N,O-bis-(trimethylsylil)-trifluoroacetamide with 1% trimethylchlorosilane and analyzed by gas chromatography-mass spectrometry (GC-MS) on a SHIMADZU GC-17A/QP-5050, equipped with a J&W DB-1 capillary column (25 m×0,2 mm×0,33 µm). The following GC-MS parameters were used: 1 ml/min (helium) flux; split ratio, 1∶10; injector temperature 250°C; detector temperature 270°C; temperature gradient: 100°C to 270°C in 33 min, peak detection method: selected ion monitoring technique [8]. All solvents, reagents, and standards of α-tocopherol, cholesterol, and squalene for GC-MS analysis were from Sigma-Aldrich (Milan, Italy).

### Squalene isolation from skin surface lipids

Measured aliquots of SSL samples were separated by thin layer chromatography (TLC) with benzene/hexane (70/30) elution system, with commercial naïve and irradiated Sq (Sigma-Aldrich) as standards. The Sq fraction was extracted quantitatively with chloroform/methanol (2/1) from the silica spots of the SSL lane (Fig. S1), thoroughly filtered, evaporated and diluted in minimal quantity of methanol. To verify the purity of isolated Sq, the fraction was subjected to GC-MS analysis, and the typical spectrum (Fig. S2) showed more than 97% purity.

### Cell cultures

Primary cultures of normal human epidermal keratinocytes (NHEK) were obtained from skin biopsies of healthy volunteers (n = 5) after their informed consent [30]. Keratinocytes were grown up to 60–80% confluence in serum-free medium, supplemented with hydrocortisone, EGF, insulin, epinephrine, transferrin, bovine pituitary extract, and gentamycin/amphotericin (KGM-Gold, Lonza, Walkersville, MD). In the 24 h preceding experiments, NHEK cultures were switched to supplement-depleted medium.

### Irradiation of cells and skin surface lipids with solar simulated UVA and UVB

NHEK monolayer was exposed to low-dose UVA+UVB irradiation (time of irradiation 30 s, distance from cells 30 cm, dose UVA 1.0 J/cm^2^+UVB 0.1 J/cm^2^) produced by Solar Simulator (Dermalight Vario with filter A2, Dr. Hoehnle AG, UV Technology, Planegg, Germany) with emission spectrum from 280 nm and emission peak at 375 nm. The light effluence rate on the cell monolayer was 40 mW/cm^2^.

In the *in vivo* experiments, dorsum skin of 10 healthy donors was exposed to the same UVA+UVB lamp (the distance 80 cm, irradiation time 30 min, dose UVA 30.0 J/cm^2^+UVB 3.0 J/cm^2^) and SSL were immediately collected and analyzed.

In the *ex vivo* experiments, dried-under-nitrogen SSL extracts (0,95 mg) were placed to glass Petri dishes (diameter 2 cm), and irradiated by the same UVA+UVB source (the distance from the bottom of dishes was 30 cm, irradiation time range 1–20 min, and dose range from UVA 1.0 J/cm^2^+UVB 0.1 J/cm^2^ to UVA 40.0 J/cm^2^+UVB 4.0 J/cm^2^). Immediately after irradiation, SSL extracts were dissolved in chloroform/methanol (2∶1) with the addition of butylated hydroxyanisole to stop lipid peroxidation, and analyzed quantitatively for α-tocopherol, cholesterol, and squalene levels. Results of triplicate experiments were expressed as percent amount of α-tocopherol, squalene, or cholesterol in SSL as compared to non-irradiated controls. The same irradiation procedure was applied to commercial Sq, to use it as a reference standard of irradiated squalene for TLC and in the experiments with NHEK.

### NHEK exposure to substances mediating UV effects

To simulate UV effects on AhR controlled metabolic pathways, 6-formylindolo[3,2-b]carbazole (FICZ, 0.1 µM and 1 µM, Biomol Research, Plymouth Meeting, CA), known to be an endogenous mediator of UV-induced signal transduction to AhR-driven machinery [11], [34], was added to NHEK cultures for 1 h.

In the experiments with 4-HNE (Cayman Chem, Ann Arbor, MI), reported to mediate UVB signaling to EGFR pathway in keratinocytes [27], [35], a 25 µM 4-HNE solution in DMSO or vehicle alone were added to NHEK. After 0.5–4 h of incubation, the conditioned medium was collected for further cytokine assay, while cells were processed for total lysate preparation and RNA isolation.

To elucidate possible SSL effects as sensors/mediators of solar UV signals to skin cells, NHEK cultures were exposed to the lipids normalized by the content of Sq, or SqPx isolated from *ex vivo* irradiated SSL, or SqPx-*in vivo* isolated from *in vivo* irradiated SSL (final concentrations of Sq, PxSq, or SqPx-*in vivo* were 1.0 µg/mL).

The effects of *tert*-butylperoxide and photo-oxidized linoleic acid (both from Sigma-Aldrich, Milan, Italy) were compared to the effects of photo-oxidized SSL and photo-oxidized Sq. *Tert*-butylperoxide was added to NHEK at two concentrations (0.1 mg/mL and 0.5 mg/mL). Linoleic acid was irradiated by UVA+UVB using the same protocol as for SSL, and the products of its photo-oxidation were added to NHEK at final concentrations 0.1 µg/mL and 0.4 µg/mL, the latter corresponds to the physiological ratio of squalene:linolenic acid % concentrations in SSL equal to 10∶1 [2], [7]. The concentration of photo-oxidized linoleic acid 0.4 µg/mL corresponds to its 2 µM. The same molar concentration of PxSq was used in all experiments.

### Transfection with AhR- specific small interference RNA

AhR was knocked-down by using a pool of four small interfering RNAs (siRNAs) provided by Euroclone (Milan, Italy) (ONTARGET*plus* SMARTpool, L-004990–00–0005). In parallel, a pool of four nontargeting siRNAs was used as negative control (ON-TARGET *plus* siCONTROL, D-001810–10–05). NHEK were incubated with a mixture of 50 nM SiRNA and 4 µL/mL INTERFERIN™ transfection reagent (Polyplus Transfection, Euroclone), according to the manufacturer's instructions. After 48 h incubation in starvation medium, SiRNA-transfected cells were exposed to FICZ for the indicated time interval. Cells were then lysed for protein extraction, or for RNA extraction.

### Preparation of cell extracts and immunoblotting

Total non-phosphorylated and phosphorylated EGFR, ERK1/2, Akt1, and phosphorylated p65 subunit of NFκB were investigated in total cell lysates as previously described [36]. Anti-EGFR antibodies were from Santa Cruz, whereas anti-ERK, anti-Akt1, and anti-p65 NFκB antibodies were from Cell Signaling Technology (Beverly, MA).

NHEK were differentially lysed to obtain nucleic extracts according to [37]. The antibodies used for the detection of AhR, Arnt, non-phosphorylated and phosphorylated EGFR were from Santa Cruz Biotechnology, CA.

### RNA isolation and quantitative real-time-PCR assay

Total RNA was isolated using the GenElute Mammalian Total RNA Kit (Sigma-Aldrich, Milan, Italy) and was reverse-transcribed using the iScript cDNA Synthesis Kit (Bio-Rad, Hercules, CA). cDNA was amplified with IQ SYBR green Supermix (Bio-Rad, Hercules, CA), using the MiniOpticon Real-Time PCR Detection System (Bio-Rad, Hercules, CA). Two housekeeping genes, ribosomal 18S and beta-actin were chosen as reference, and fold changes were calculated [38]. The primer sets were synthesized by Eurofins MWG Operon (Ebersberg, Germany):

*β-actin* fwd:5′-AATCTGGCACCACACCTTCTAC-3′; *β-actin* rev:5′-ATAGCACAGCCTGGATAGCAAC-3′; *18S* rRNA fwd:5′- TCCCCCAACTTCTTAGAGG-3′; *18S* rRNA rev:5′- GCTTATGACCCGCACTTAC-3′; *COX-2* frw:5′-TTCTCCTTGAAAGGACTTATGGGTAA-3′; *COX-2* rev:5′-AGAACTTGCATTGATGGTGACTGTTT-3′ *; CYP1A1* frw:5′-CCTGGAGACCTTCCGGCACT-3′ ; *CYP1A1* rev:5′-AGACACAACGCCCCTTGGGG-3′ ;*CYP1B1* frw:5′-TGGTCTGTGAATCATGACCCAGTGA-3′; *CYP1B1* rev:5′-TCTTCGCCAATGCACCGCCT-3′; *G2A* frw: 5′-CGCACAGAGACAAAGTGGAA-3′; *G2A* rev: 5′-CTCATCTTCCCAAACGGAGA-3′; *IL1β* fwd:5′-TGGCTCATTTTCCCTCAAAAGTTG-3′; *IL1β* rev:5′-AGAAATCGTGAAATCCGAAGTCAAG-3′; *IL-6* fwd:5′-GTGTGAAAGCAGCAAAGAG-3′; *IL-6* rev:5′-CTCCAAAAGACCAGTGATG-3′; *IL-8* frw:5′-GTCCTTGTTCCACTGTGCCT-3′; *IL-8* rev:5′-GCTTCCACATGTCCTCACAA-3′; *IL-1R1* frw: 5′-CCAGAGGTCAGGAGTTCGAG-3′; *IL-1R1* rev: 5′-CCACCATGCCTAGCTCATTT-3′; *iNOS* fwd:5′- TACTCCACCAACAATGGCAA-3′ *; iNOS* rev:5′- ATAGCGGATGAGCTGAGCAT-3′; *MCP1* frw:5′-AAGCAGAAGTGGGTTCAGGA -3′; *MCP1* rev:5′-TAAAACAGGGTGTCTGGGGA-3′; *MYD88* fwr: 5′-GAGCCTAACCATGTCCCTGA-3′; *MYD88* rev: 5′-TGGGTCCTTTCCAGAGTTTG-3′; *TLR4* fwd: 5′-AGTCCATCGTTTGGTTCTGG-3′; *TLR4* rev: 5′-CAATGGTCAAATTGCACAGG-3′; *TNFα* fwd:5′- TCCTTCAGACACCCTCAACC-3′; *TNFα* rev: 5′- AGGCCCCAGTTTGAATTCTT-3′.

### Assays for inflammatory cytokine production by NHEK

The pro-inflammatory cytokines TNFα, IL-6, and IL-8 were measured in cell supernatants at 24 h post-irradiation or SqPx exposure, using BD OptEIA Elisa kits from BD Biosciences (San Diego, CA) [36].

### Statistics

All measurements were done in triplicate, and data of at least three independent experiments were statistically evaluated. Statistical evaluation was carried out with the software package for Windows XP. Results were expressed as the mean ±SD. To evaluate the difference between experimental groups, the two-tailed Student's *t*-test was applied and *P* values<0.05 were considered to be significant.

## Results

### Physiological doses of solar simulated UVA+UVB induce inflammatory responses time dependently in NHEK

**Figure 1:**
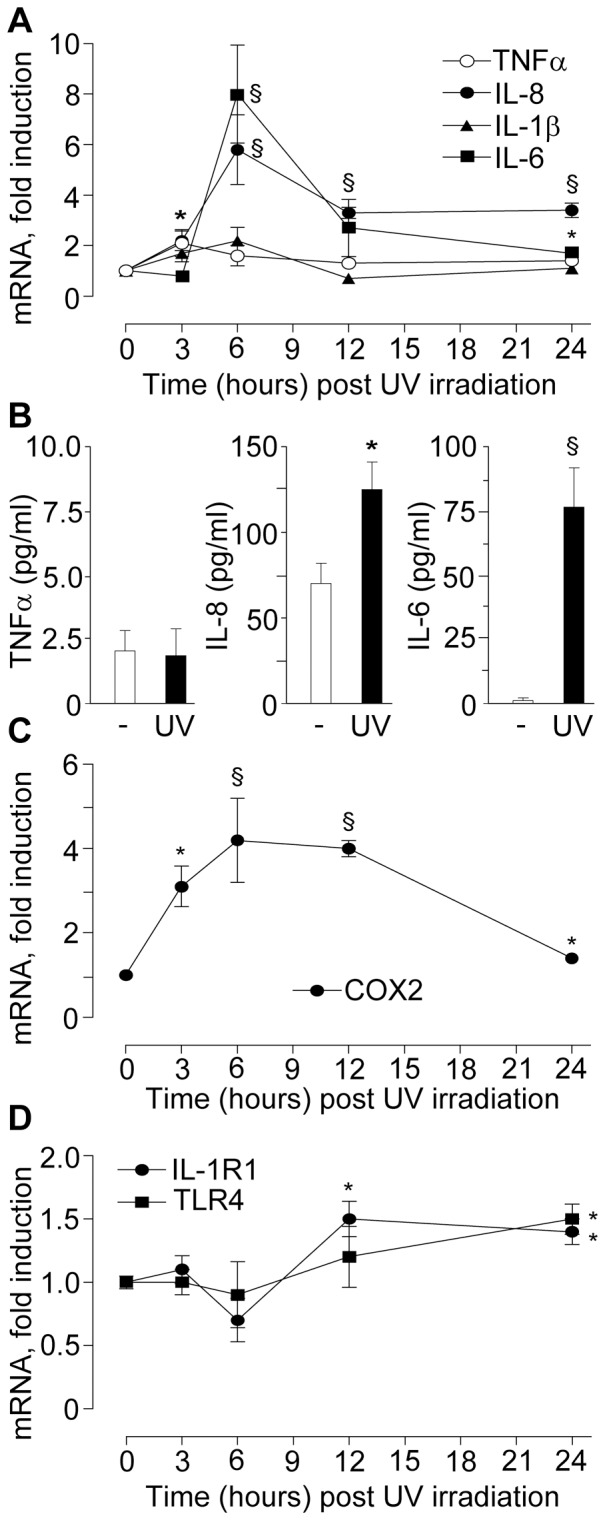
Inflammatory responses to UVA+UVB in normal human epidermal keratinocytes (NHEK). **A**. Time course of post-UV cytokine transcripts; **B**. TNFα, IL-6 and IL-8 proteins in the conditioned medium of sham-irradiated NHEK and 24 h post UVA+UVB irradiation (1.0+0.1 mJ/cm^2^); **C**. Time course of post-UV *COX-2* transcript; **D**. Time course of post-UV *IL-1R1* and *TLR4* transcripts. *P<0.05 and ^§^P<0.01*versus* sham-irradiated control.

Exposure of NHEK to UVA+UVB led to time-dependent bell-shaped induction of genes encoding the pro-inflammatory cytokines *TNFα*, *IL-8*, and *IL-1β* and delayed induction of *IL-6* (Fig. 1A). Maximal although slight expression of *TNFα* and *IL-1β* was reached at 3 h, while the other pro-inflammatory cytokines were strongly and maximally expressed at 6 h. The protein level of TNFα did not change, whereas those of IL-8 and IL-6 were significantly increased in NHEK supernatants at 24 h post-irradiation (Fig. 1B). At 6 h, maximal expression of *COX-2* mRNA was observed (Fig. 1C). The mRNA expression of the two membrane receptors *TLR4* and *IL-1R1* was first temporarily suppressed and then, gradually increased up to 12–24 h (Fig. 1D).

### Physiological doses of solar simulated UVA+UVB activate AhR system and downstream cytochrome P450 isoforms CYP1A1 and CYP1B1

**Figure 2:**
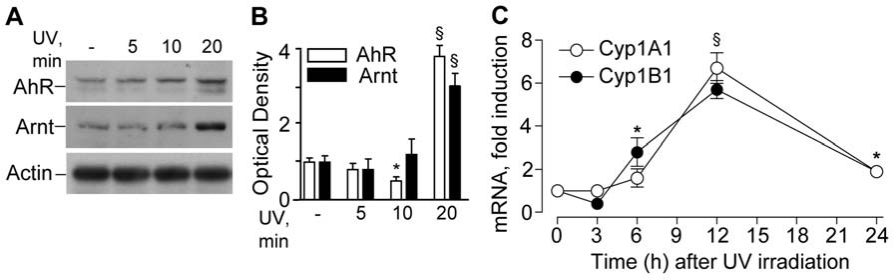
Time-dependent effects of UV irradiation on metabolic system in NHEK. **A**. Time-dependent effects on nuclear levels of AhR and Arnt post-UV irradiation. Actin was used as a loading control; **B**. Densitometry of western blot bands of AhR and Arnt in the nucleus; *P<0.05 and ^§^P<0.01 *versus* untreated controls (-). **C**. Time-course of CYP1A1 and CYP1B1 transcripts post-irradiation; *P<0.05 and ^§^P<0.01 *versus* untreated controls (0 h). Significance refers to both the transcripts at each time-point. The results are representative of three independent experiments.

**Figure 3:**
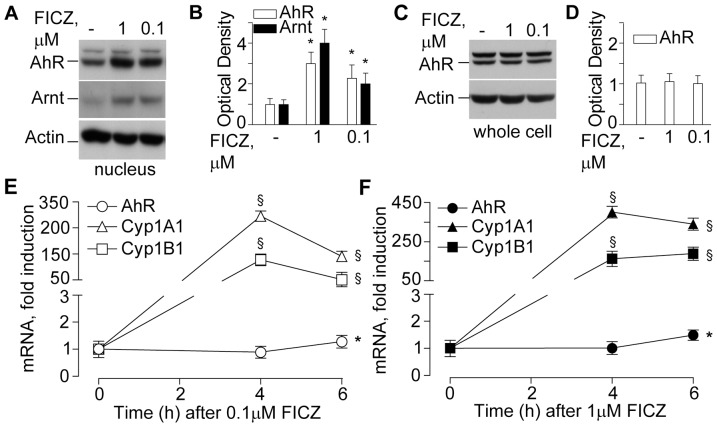
Effects of FICZ on metabolic system in NHEK. **A**. Western blots of nuclear AhR and Arnt after 30 min exposure to 0.1 or 1.0 µM FICZ. **B**. Densitometry of western blot bands. **C**. Western blots of total whole cell AhR after 30 min exposure to 0.1 or 1.0 µM FICZ. **D**. Densitometry of western blot bands. *P<0.05 and §P<0.01 *versus* untreated controls (-). Time-course of AhR, CypA1 and Cyp1B1 levels following treatment with 0.1 µM FICZ (**E**) and 1 µM FICZ (**F**). *P<0.05 and §P<0.01 *versus* untreated controls (0 h). The results are representative of three independent experiments.

**Figure 4:**
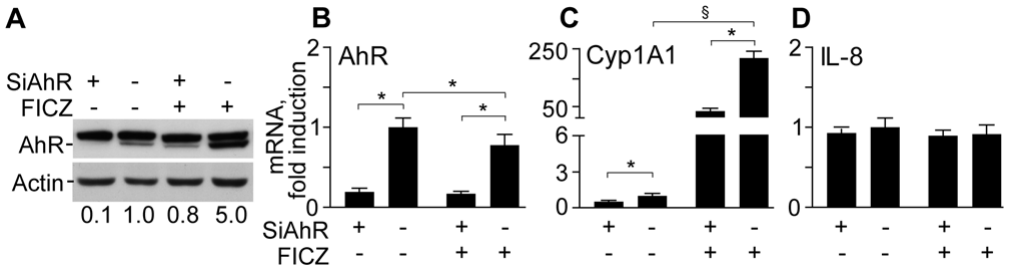
Effects of AhR silencing (SiAhR) on metabolic system and *IL-8* expression in NHEK. A. AhR levels in NHEK treated for 24 h with 1 µM FICZ. The treatment was started 48 h after initiation of RNA silencing of AhR (SiAhR) or mock (-). The mean values of the densitometry of AhR bands (three independent determinations) are reported. Levels of *AhR* (B), *Cyp1A1* (C) and *IL-8* (D) transcripts in NHEK treated for 24 h with 1 µM FICZ. *P<0.05 and ^§^P<0.01.

UVA+UVB irradiation stimulated AhR and Arnt nuclear translocation (Figs. 2A and 2B), an early event preceding the induction of *CYP1A1* and *CYP1B1* genes, which reached maximal levels 12h post-irradiation (Fig. 2C). AhR and Arnt nuclear translocation was significantly and dose-dependently triggered also by the product of tryptophan photo-oxidation FICZ (Figs. 3A and 3B). At the same time, total AhR protein level were not affected by FICZ (Figs. 3C and 3D), implying that the aromatic compound is an AhR ligand, which did not induce its *de novo* synthesis, but rather re-distributed the cytoplasmic receptor between nucleus and cytoplasm. Accordingly, both *CYP1A1* and *CYP1B1* were also strongly, dose-dependently up-regulated by FICZ, with maximal effect already achieved at 4 h (Figs. 3E and 3F). In addition, FICZ slightly but statistically significantly induced delayed *AhR* expression at 6 h (Figs. 3E and 3F). Upon AhR protein level knock-down by specific siRNA (Fig. 4A), both spontaneous and FICZ-associated AhR were dramatically down-regulated as expected (Fig. 4B), and correspondingly also CYP1A1 levels were remarkably reduced (Fig. 4C). Of note, constitutive IL-8 was not affected by AhR silencing or by the presence of FICZ (Fig. 4D). Similarly, FICZ did not perturb the expression of other pro-inflammatory genes, including TNFα, IL-6, and COX-2, the induction of which is characteristic of NHEK response to UV [33].

### Physiological doses of solar-simulated UVA+UVB stimulate signal transduction through EGFR phosphorylation, downstream cytoplasmic signal transduction, and EGFR translocation to the nucleus

**Figure 5:**
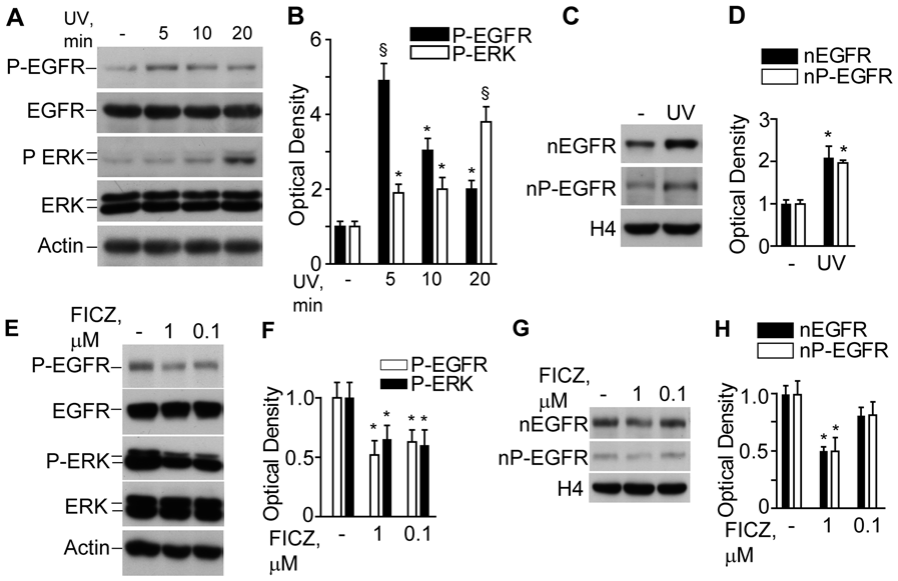
Effects of UV and FICZ on the EGFR signalling in NHEK. **A**. Western blot detection of the phosphorylated forms of EGFR (P-EGFR) and ERK (P-ERK) in total lysates of untreated NHEK (-) and post-UV irradiation; **B**. Densitometry of the bands. *P<0.05 and ^§^P<0.01 *versus* untreated controls (-). **C**. Western blots of nuclear non-phosphorylated (nEGFR) and phosphorylated EGFR (nP-EGFR) before and 20 min after exposure to UV. Histone 4 (H4) was used as a loading control; **D**. Densitometry of nEGFR and nP-EGFR bands. *P<0.05 *versus* untreated control (-). **E**. Western blot detection of the phosphorylated forms of EGFR (P-EGFR) and ERK (P-ERK) in total lysates of untreated NHEK (-) and after 30 min exposure to 0.1 or 1.0 µM FICZ. **F**. Densitometry of the bands. *P<0.05 *versus* untreated control (-). **G**. Western blots of nuclear non-phosphorylated (nEGFR) and phosphorylated EGFR (nP-EGFR) after 30 min exposure to 0.1 or 1.0 µM FICZ; **H**. Densitometry of nEGFR and nP-EGFR bands. *P<0.05 *versus* untreated controls.

NHEK exposure to UV led to a rapid (5 min) and transient EGFR phosphorylation, while downstream ERK phosphorylation gradually increased up to 20 min post-irradiation when examined in total cell lysates (Figs. 5A and 5B). At 20 min post-irradiation, nuclear levels of both non-phosphorylated and phosphorylated EGFR were significantly increased (Figs. 5C and 5D). Conversely, exposure to FICZ did inhibit both EGFR and ERK in a dose-dependent fashion (Figs. 5E and 5F). Moreover, 1 µM FICZ significantly decreased nuclear levels of both non-phosphorylated and phosphorylated EGFR (Figs. 5G and 5H).

### Exogenous 4-HNE dysregulates EGFR-connected signal transduction pathways time dependently, induces COX-2 expression but it only slightly affects pro-inflammatory cytokines and iNOS

**Figure 6:**
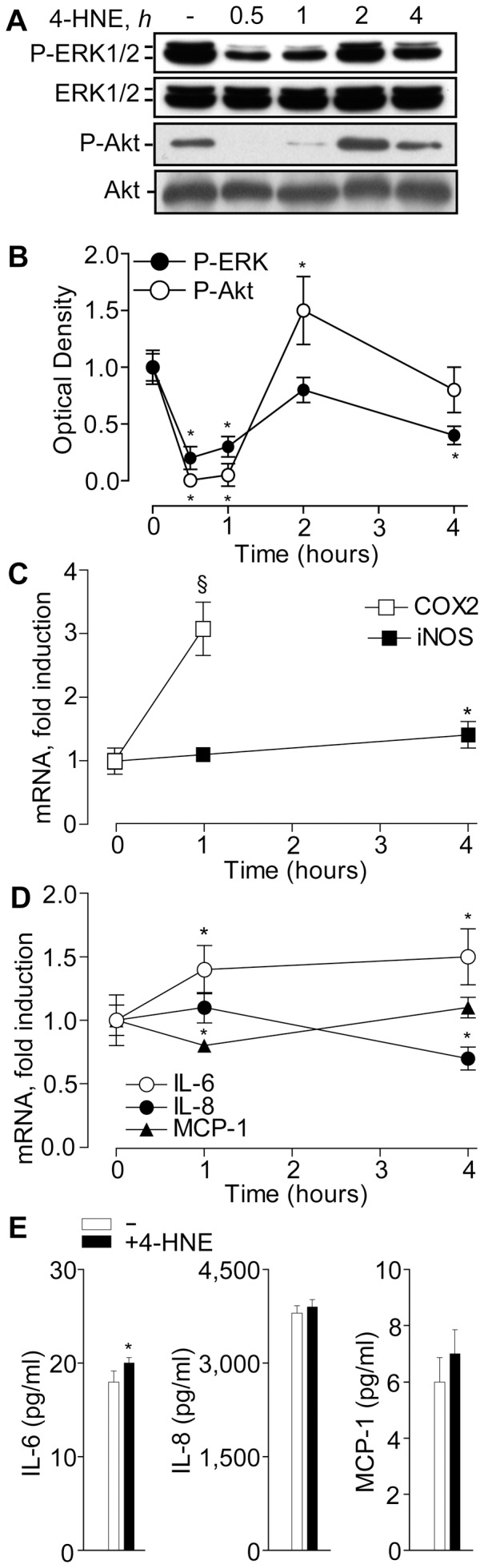
4-hydroxy-2-nonenal (HNE) effects on signal transduction and expression of pro-inflammatory mediators in NHEK. Time-dependent effects of 25 µM HNE on ERK1/2 and Akt1 phosphorylation: **A**. Western blot and **B**. Densitometry of total cell extracts. Dynamics of HNE effects on COX-2 and iNOS (**C**) and pro-inflammatory cytokine (**D**) gene expression. Cytokine levels in the culture medium after 24 h treatment with 25 µM HNE (**E**). *P<0.05 and ^§^P<0.01 *versus* untreated controls.

We observed that a non-toxic dose of 4-HNE (25 µM), known to stimulate EGFR nuclear translocation and to mimic UVB effects on primary human keratinocyte cultures [29], inhibited downstream ERK and affected Akt1 phosphorylation in a bi-phasic fashion, with transient inhibition at 0.5 and 1 h, but stimulation at 2 h (Figs. 6A and 6B). With regard to inflammatory gene response, exposure to 4-HNE induced rapid and significant *COX-2* expression, slight and delayed *iNOX* (Fig. 6C), and durable although slight *IL-6* expression (Fig. 6D). None of the other EGFR-connected pro-inflammatory chemokines, *IL-8* or *MCP-1* or *IP-10* (data not shown), were affected by 4-HNE. Protein expression of IL-6, IL-8, and MCP-1 corresponded to their mRNA behaviour (Fig. 6E).

### Squalene derivatives isolated from photo-oxidised skin surface lipids but not intact lipids/squalene induce UV-similar mRNA pattern of pro-inflammatory cytokines and receptors

**Figure 7:**
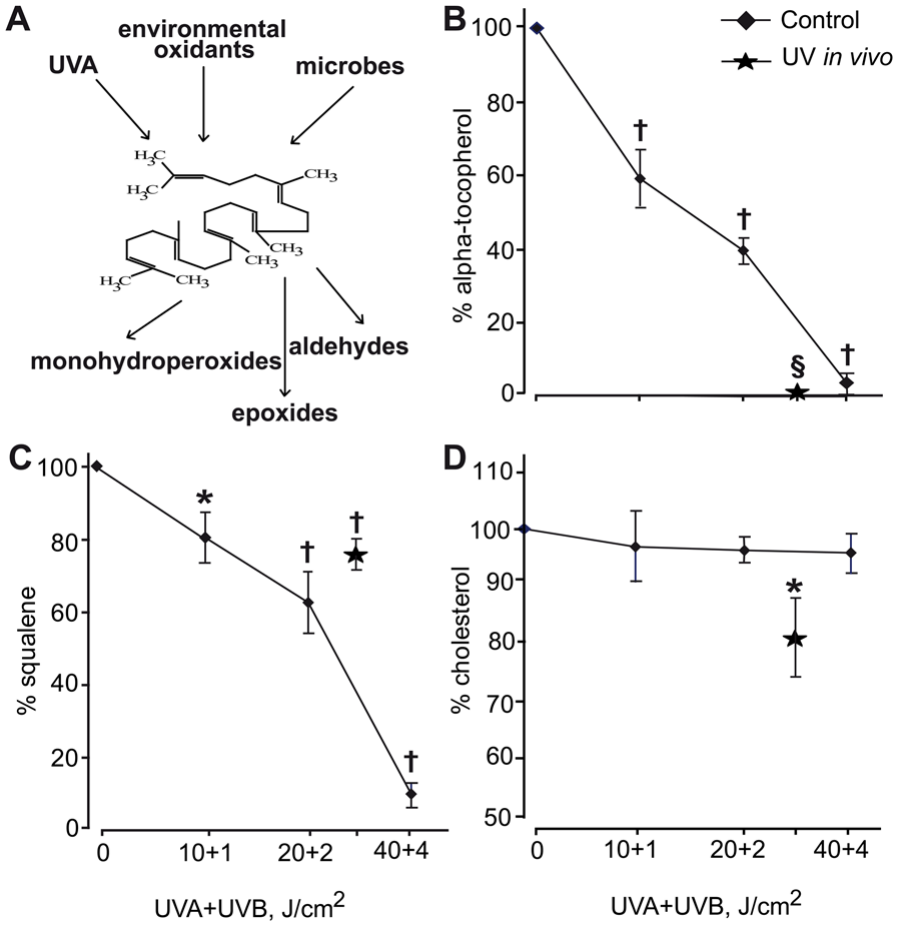
Degradation of UV-sensitive components of human skin surface lipids (SSL). SSL were *ex vivo* exposed to increasing doses of solar-simulated UV irradiation or *in vivo* exposed to cumulative dose of UVB+UVA = 3+30 J/cm^2^. **A**. Scheme of SSL squalene oxidation; dose-dependent (UVA+UVB, J/cm^2^) degradation of oxidisable SSL fractions **B**. Squalene; **C**. α-Tocopherol; **D**. Cholesterol. Stars represent results of *in vivo* exposure of healthy skin (n = 10) to solar-simulated UV light. *P<0.05 and ^†^P<0.01 *versus* non-irradiated controls.

Freshly collected SSL were exposed *ex vivo* to solar simulated low dose UVA+UVB irradiation for 5–20 min, and the content of UV-sensitive (α-tocopherol and Sq) and UV-insensitive (cholesterol) substances was monitored. *In vivo* skin irradiation with UVA+UVB (30+3 J/cm^2^) also resulted in the degradation of photo-oxidisable components of SSL (Fig. 7B–7D).

Intact SSL, SSL irradiated *ex vivo* for 20 min (oxSSL), Sq isolated from intact SSL and from oxSSL (SqPx), and from *in vivo* irradiated SSL (SqPx-*in vivo*), as well as commercial Sq photo-irradiated for 20 min (Fig. S1), were normalised by Sq or SqPx content (1 µg/mL) and added to NHEK cultures. The purity of SqPx levels isolated from *ex vivo* or *in vivo* irradiated SSL was >99% (Fig. S2), and the levels of Sq or SqPx in SSL or oxSSL, respectively, ranged from 10 to 15%.

**Figure 8:**
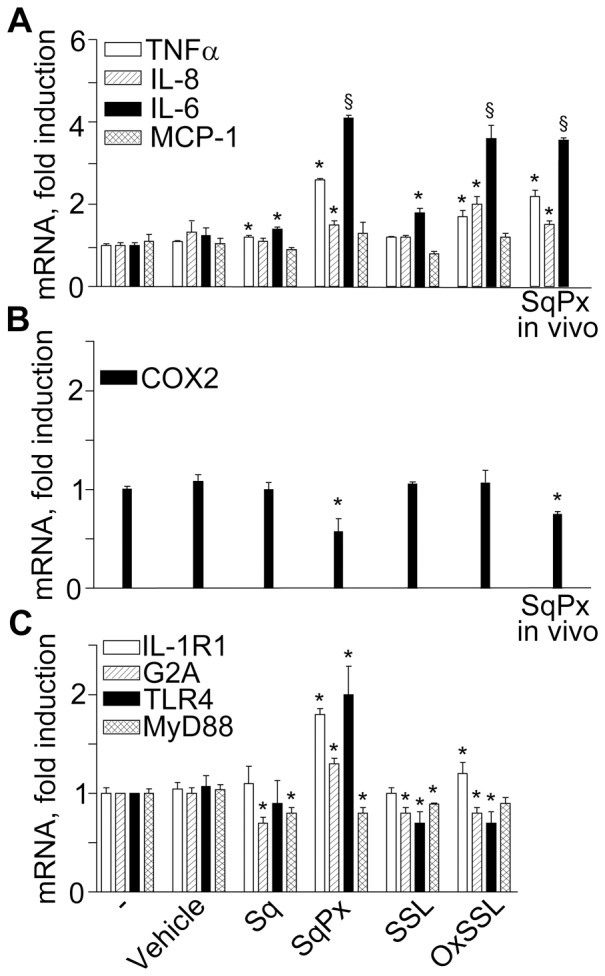
Effects of skin surface lipids on gene expression in NHEK. NHEK cultures were treated with skin surface lipids normalised by the content of squalene (Sq): intact freshly prepared skin surface lipids (SSL, with final Sq content 1 µg/mL), Sq isolated from the SSL (final content 1 µg/mL), SSL exposed to UVA+UVB for 20 min (oxSSL with final content of oxidised Sq (SqPx) 1 µg/mL), or PxSq isolated from oxSSL (final content of SqPx 1 µg/mL) or SqPx-*in vivo* isolated from the in vivo photo-exposed skin surface lipids (final content of SqPx-*in vivo* 1 µg/mL). The panels present fold of mRNA changes for pro-inflammatory cytokines (**A**), *COX-2* (**B**), and membrane receptors (**C**). *P<0.05 and ^§^P<0.01 *versus* control treated with the vehicle for the lipids (0.1% methanol).

Fig. 8A shows that co-incubation of NHEK with intact SSL for 3 h practically did not affect gene expression of pro-inflammatory cytokines (*TNFα*, *IL-8*, and *MCP-1*) and only slightly induced *IL-6*. In addition, SSL did not exert any effect on *COX-2* (Fig. 8B) and *IL-1R1* mRNA, while it slightly down-regulated *G2A*, *TLR4* and its downstream co-activator *MYD88* (Fig. 8C). Sq isolated from intact SSL exhibited similar mode of action. On the other hand, oxSSL up-regulated *TNFα*, *IL-6*, and *IL-8*, but it did not change *MCP-1* (Fig. 8A) or *COX-2* (Fig. 8B), *IL-1R1*, and *MYD88* expression, while it slightly inhibited *G2A* and *TLR4* (Fig. 8C). SqPx strongly up-regulated *TNFα*, *IL-6*, *IL-8*, *IL-1R1*, *G2A*, and *TLR4* (Fig. 8A and 8C), although it down-regulated *COX-2* and *MYD88* (Fig. 8B and 8C). SqPx-*in vivo* up-regulated *TNFα, IL-6, IL-8*, while down-regulating *COX-2* (Fig. 8A and 8B). The behaviour of commercial Sq upon UVA+UVB exposure was similar to SqPx (Table 1). It could be, however, noticed that photo-oxidized commercial Sq was less effective than Sq isolated from SSL exposed to the same dose of UVA+UVB. This could be explained by the fact that some lipids in SSL were co-oxidized with Sq thus accelerating its photo-oxidation. Also, trace amounts of transition metals, such as Fe and Cu, in SSL could catalyze photo-oxidation of Sq. Therefore, either more SSL squalene was photo-oxidized or deeper oxidation of SSL Sq (different oxidation metabolites) occurred as compared to pure squalene.

**Table 1:** Induction of inflammatory and metabolic genes (mRNA, fold of induction) by photo-oxidized commercial squalene and squalene from photo-oxidized human skin surface lipids (SSL).

Genes	Squalene isolated from photo-oxidized SSL	Photo-oxidized commercial squalene
*TNF-alpha*	2.5±0.10^*^	1.9±0.10^*^
*IL-6*	1.4±0.03^*^	1.6±0.06^*^
*IL-8*	1.5±0.12^*^	1.7±0.10^*^
*CYP1A1*	8.3±0.06^†^	4.2±0.26^†^

Commercial squalene or SSL were exposed to UVA+UVB irradiation (dose UVA = 20 J/cm^2^ and UVB = 2 J/cm^2^). Photo-exposed SSL were subjected to TLC and squalene containing spot was collected as per Materials and Methods. Squalene samples or 0.1% methanol (a vehicle for squalene) were added to NHEK cultures (final concentration - 1 µg/mL) for 3 h. Then, cells were thoroughly washed and RNA was isolated. Gene expression was determined by RT-PCR and results were expressed as a fold of induction versus vehicle. Three independent experiments were carried out and the mean values ±S.D. were calculated. *P<0.05 and ^†^P<0.001.

To evaluate whether hydroperoxides of photo-oxidized linoleic acid, which is a minor oxidizable component of human SSL [2], [7], could possess solar UV-mimicking effects similar to PxSq, photo-oxidized commercial linoleic acid was added to NHEK in physiologically relevant concentrations (0.1 and 0.4 µg/mL). Contrary to UV and PxSq, photo-oxidized linoleic acid inhibited *TNFα*, *IL-6*, *IL-8*, and *IL1R1*. Stimulation of *IL-8* expression occurred upon NHEK exposure to 0.4 µg/mL of photo-oxidized linoleic acid (Table 2). *Tert*-butyl hydroperoxide, a synthetic organic hydroperoxide traditionally used to model UVA effects on skin cells, at millimolar concentrations induced *TNFα*, *IL-8*, and *IL1R1* while suppressed *IL-6* (Table 2).

**Table 2:** Effects of photo-oxidized linoleic acid and *tert*-buthyl hydroperoxide (tBOOH) on the expression of solar UV-sensitive genes in cultivated primary human keratinocytes.

GENES	*TNF*α	*IL-6*	*IL-8*	*CYP1A1*	*CYP1B1*	*IL1R1*
SYSTEM						
**Control**	1.00±0.22	1.03±0.25	1.00±0.06	1.00±0.03	1.00±0.04	1.0±0.1
**tBOOH, 0.1 mg/mL**	14.70±0.36^#^	0.79±0.03	3.10±0.15^&^	0.01±0.00^#^	0.04±0.01^#^	1.40±0.03*
**tBOOH, 0.5 mg/mL**	25.60±0.85^#^	0.53±0.06	2.70±0.06^&^	0.01±0.00^#^	0.01±0.01^#^	1.30±0.06*
**Photo-oxidized linoleic acid, 0.1 µg/mL**	0.30±0.05*	0.61±0.01	0.40±0.06^#^	0.57±0.04^#^	0.81±0.02^&^	0.90±0.04
**Photo-oxidized linoleic acid, 0.4 µg/mL**	0.7±0.1	0.83±0.06	2.80±0.06^#^	0.20±0.02^#^	0.39±0.01^&^	0.70±0.00*

NHEK were incubated with photo-oxidized (see Materials and Methods) linoleic acid or with *tert*- butyl hydroperoxide (tBOOH) or with their vehicle methyl alcohol (0.02%, control) for 3 hours. Gene expression was determined by real-time PCR and results (fold of gene expression) are espressed as mean values ±S.D. of three independent experiments. *P<0.05, ^&^P<0.01, and ^#^P<0.001 versus control.

### Skin surface lipids profoundly inhibit EGFR-ERK axis and activate AhR system, but exclusively SqPx up-regulates cytochrome P450 isoforms (CYP1A1 and CYP1B1)

**Figure 9:**
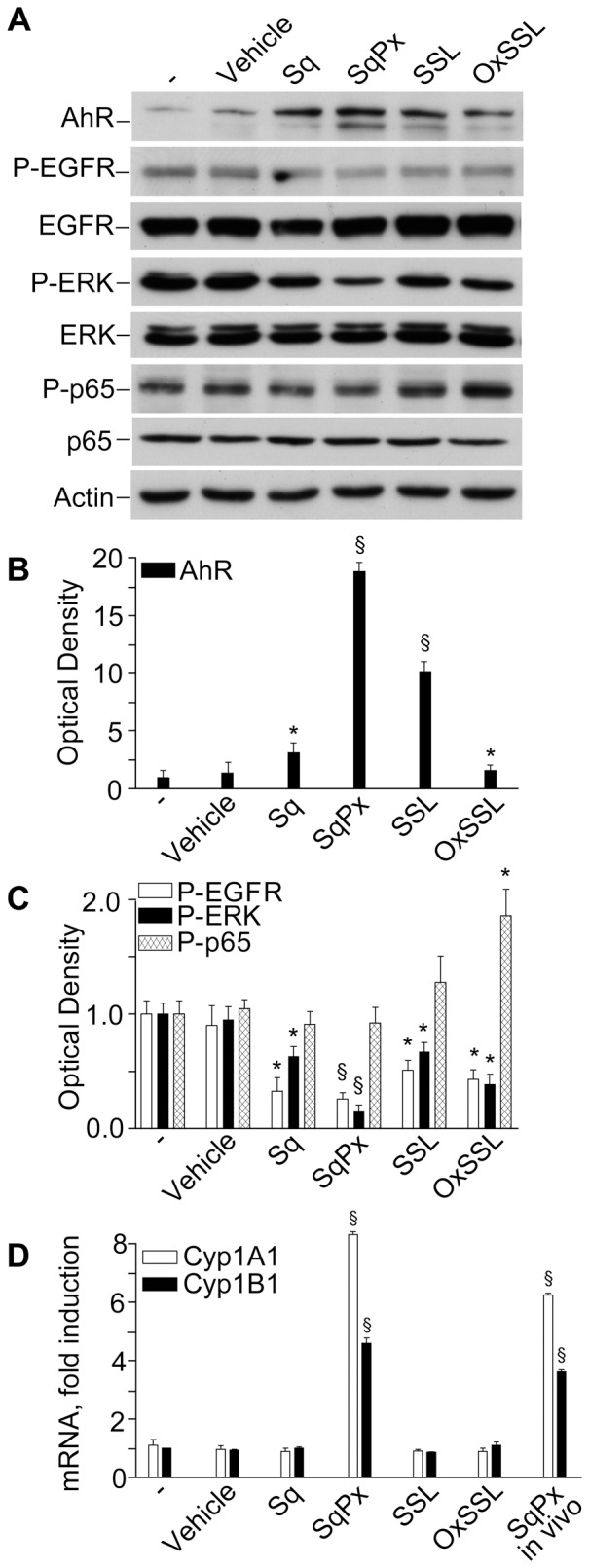
Effects of intact and UV-exposed skin surface lipids on signal transduction and AhR-CYP1 axis in NHEK. NHEK cultures were exposed to skin surface lipids normalised by the content of squalene (Sq): intact freshly prepared skin surface lipids (SSL, with final Sq content 1 µg/mL), Sq isolated from the SSL (final content 1 µg/mL), SSL exposed to UVA+UVB for 20 min (oxSSL with final content of oxidised Sq (PxSq) 1 µg/mL), or SqPx isolated from oxSSL (final content of SqPx 1 µg/mL), or SqPx-*in vivo* isolated from the in vivo photo-exposed skin surface lipids (final content of SqPx-*in vivo* 1 µg/mL). After 20 min of co-incubation, differential lysis was carried out, cytoplasmic and nuclear proteins extracted, and subjected to electrophoresis (**A**). Western blots were quantified by densitometry: (**B**) nuclear levels of AhR and (**C**) cytoplasmic levels of phosphorylated EGFR (P-EGFR), ERK (P-ERK), and the NFκB constituent p65 (P-p65). Transcripts of *CYP1A1* and *CYP1B1* were determined by RT-PCR at 3 h co-incubation (**D**). *P<0.05 and ^§^P<0.01 *versus* control treated with the vehicle for the lipids (0.1% methanol).

All forms of skin surface lipids studied activated AhR translocation to the nucleus and the effect changed in the range: SqPx>SSL>oxSSL = Sq (Figs. 9A and 9B). Surprisingly, only SqPx and SqPx-*in vivo* up-regulated *CYP1A1* and *CYP1B1* genes, downstream targets for AhR (Fig. 9D). Both photo-oxidized linoleic acid and *tert*-butyl hydroperoxide significantly down-regulated *CYP1A1* and *CYP1B1* genes (Table 2). Several recent publications [34] have explained the initial transient inhibition of AhR nuclear translocation and *CYP1* expression by early transient activation of NFκB. Indeed, p65 phosphorylation was significantly induced only by oxSSL (Fig. 9C), while it did not stimulate *AhR* (Fig. 9B) or *CYP1* gene expression (Fig. 9D). The SSLs were also effective inhibitors of EGFR and ERK phosphorylation, SqPx being the strongest (Figs. 9A and 9C).

## Discussion

Mechanisms of solar UV irradiation effects towards human skin have been the field of intense research mostly targeting UV-associated carcinogenesis and premature skin ageing [1], [5], [15], [38]. Based on the different capability of UVB and UVA light to penetrate in the epidermal and dermal layers, most of the studies have been focused on the endogenous photo-receptors localised in the viable cellular compartments of the skin, such as DNA, urocanic acid, melanin precursors, porphyrins, tryptophan, dihydropyridins, etc. [2], [14], [39]. These cell-located molecules selectively absorb UV light within a distinct wavelength range and, in the presence of oxygen, initiate oxidative photo-reactions affecting cell signalling and enzymatic activities, which result in characteristic UV responses of the skin cells and UV-induced changes of extracellular skin matrix.

Lipid skin surface mantel represents the first-line protective barrier between solar UV and viable human skin structures: it directly and very effectively absorbs UVB and UVA rays [2], [5], inhibits free radical-driven lipid peroxidation (α-tocopherol and coenzyme Q_10_) [1]–[4], and scavenges reactive oxygen species such as singlet oxygen (squalene) [6]. Here, we confirmed that at least two UV-sensitive SSL constituents, α-tocopherol and Sq, were dose-dependently degraded during exposure to solar-simulated UVA+UVB (Fig. 7).

The products of SSL photo-oxidation, such as Sq monohydroperoxides and epoxides (collectively, SqPx), aldehydes (Fig. 7A), and 9-HODA may easily diffuse in the skin depth reaching viable keratinocytes and melanocytes beneath *stratum corneum* [1], [2], thus exerting their UV-like biological effects. Indeed, SqPx was shown to induce melanocyte dendricity and melanogenesis [3] and IL-6 release from HaCaT [10]. Nuclear translocation of UVB-induced EGFR in primary human keratinocytes was mediated by 4-HNE [27], [35], and a UVB-like cytokine pattern was observed upon keratinocyte exposure to 9-HODA through UVB-like expression of *G2A* receptor [31]. Comparing data on direct UV-associated (Fig. 1) and photo-oxidised oxSSL- and SqPx-induced effects (Fig. 8) related to the expression of pro-inflammatory cytokines (*TNFα*, *IL-6*, *IL-8*, and *IL-1β*) or keratinocyte membrane receptors important for cytokine production (*IL-1R1* and *TLR4*), a striking similarity could be noticed. At the same time, the *TLR4* co-activator *MYD88*, essential for downstream gene transcription [25], [40] was down-regulated by the lipids. Also, in accord to published data on *G2A* up-regulation by UVB [31] , SqPx exerted similar action (Fig. 8C). Of importance, naive SSL collected from non-photoexposed human skin, as well as Sq isolated from these lipids were practically inactive, while SqPx alone induced the highest gene up-regulation as compared to the photo-oxidised lipid mixture oxSSL containing the same content of SqPx. We hypothesised that the complex mixture may also contain components such as linoleic acid with a mode of action opposite to SqPx. Characteristically, up-regulation of the secondary cytokine *IL-6* was slower upon irradiation than upon SqPx action (temporary down-regulation at 3 h in the latter case and a 4-fold up-regulation in the former), which suggests the necessity of UV-induced oxidative signalling molecules to start *IL-6* transcription [12]. With regard to induction of *COX-2*, another marker of UV response (Fig. 1C), exclusively SqPx exhibited opposite inhibiting action (Fig. 8B). *COX-2* [11], [23], [28], [29] as well as *iNOS* [23] expression is controlled by EGFR activation and nuclear translocation. In a good agreement with these data, solar-simulated UV induced fast (maximum at 5 min) EGFR phosphorylation (Figs. 5A and 5B), followed by downstream ERK phosphorylation (gradual increase until 20 min) and nuclear translocation of both EGFR and P-EGFR (Fig. 5C and 5D). Logically, time-dependent *COX-2* induction was observed. We hypothesised that inhibitory effects of SqPx and SqPx*-in vivo* towards COX-2 could be associated with inhibited phosphorylation (Fig. 9C), followed by inhibited nuclear translocation of EGFR. On contrast, very fast *COX-2* and *iNOS* induction was associated with NHEK exposure to non-toxic dosage of 4-HNE (Fig. 6C), able to mediate UVB-induced EGFR phosphorylation and translocation to the nucleus [28], [35]. Usually, *iNOS* up-regulation becomes detectable at 6 h post UV [41]. However, in the presence of the pre-formed redox mediator (4-HNE), a statistically significant difference was seen at 4 h. At the same time, 4-HNE did not completely mimic UV in terms of inflammatory responses in NHEK, since it did not practically induce cytokines (Figs. 6D and 6E) while dramatically, although temporarily, inhibited ERK and Akt1 phosphorylation downstream EGFR (Figs. 6A and 6B).

The AhR has been suggested to be a light sensor in keratinocytes and melanocytes, since its transcriptional function and downstream CYP1 enzymes are activated by both visible and UV light, and mediated by endogenous high affinity AhR ligand FICZ, the tryptophan photo-product [11], [13], [14], [34]. Accordingly, in our hands solar-simulated UV time-dependently stimulated AhR machine (20 min, Figs. 2A and 2B), followed by CYP1A1 and CYP1B1 over-expression reaching maximum at 12 h (Fig. 2C). Similar events were induced by FICZ (Fig. 3) although maximal CYP1 expression was reached earlier (4 h). Experiments with AhR silencing showed that CYP1A1 expression was AhR-dependent (Figs. 4A and 4B), however, 1 µM FICZ slightly but significantly up-regulated AhR transcript at 6 h (Fig. 3D), but down-regulated this gene at 24 h (Fig. 4B), possibly due to negative feed-back mechanisms active in NHEK at this last time-point. Of utmost importance, we showed for the first time, that all the types of skin surface lipids studied induced nuclear translocation of AhR, although SqPx was the best, and oxSSL were the worst inducers (Figs. 9A and 9B). SqPx (approx. 1.5 µM) induced a much higher activation of AhR than FICZ (1 µM), therefore it can be regarded as a high affinity ligand for the receptor, as dioxin [13]. AhR activation resulted in an enhanced *CYP1A1/CYP1B1* expression only in the presence of SqPx (Fig. 9D). The strange co-incidence that both FICZ and SqPx also inhibited EGFR phosphorylation could be a hint on the possible involvement of EGFR in the mechanism of *CYP1A1/CYP1B1* induction. However, in our recent publication [36], we reported that resveratrol, being a strong inducer of AhR-CYP1 axis, did not suppress spontaneous or pro-inflammatory stimuli-induced EGFR phosphorylation. On these grounds, we concluded that EGFR pathway was not directly involved in the stimulation of classical AhR-CYP metabolic mechanism activated by both FICZ and SqPx. The induction of distinct inflammatory responses by SqPx could be EGFR-dependent in accord with previous publications [25], [30]. Repressed AhR and CYP1 in the presence of oxSSL corresponded to significantly activated NFκB (Fig. 9), probably induced by cholesterol oxidation products present in oxSSL [42]–[44]. Oxidative stress is known to induce NFκB pathway, whilst repressing AhR translocation and CYP1 transcription [34].

The AhR stimulation by UV initiates adaptive stress responses such as EGFR phosphorylation and internalization, and ERK phosphorylation (Figs. 5A–D) [11], *c-jun* induction [11] and cross talk with NFκB [34]. Redox regulation of UV-induced EGFR phosphorylation occurs due to oxidative inhibition of protein-tyrosine phosphatase activity [25], [28], and its nuclear translocation is controlled by 4-HNE [27], [28]. Phosphorylated EGFR binds cytoplasmic signal transduction proteins followed by activation of several pathways, such as phospholipase Cγ-protein kinase C, Ras-Raf-mitogen activated protein kinases, PI3K-Akt1, and STATs [23], [25], [30]. This complex UV-stimulated and EGFR-mediated molecular machine aims at adaptation to solar irradiation leading to cell survival and proliferation. Here, all chemical mediators of UV interaction with NHEK (FICZ, 4-HNE, and SqPx) profoundly inhibited spontaneous EGFR, ERK or Akt1 phosphorylation at 20 min. As it was previously shown by our group, deregulation of EGFR/ERK phosphorylation led to enhanced production of pro-inflammatory chemokines (IL-8, MCP-1, and IP-10) by NHEK [30], [33], [36]. Since the environmental contaminant benzo(a)pyrene [45] and plant-derived stilbenoids [36] induce redox-controlled IL-8 production *via* the AhR signalling pathway, we suggested the same mechanism for *IL-8* induction by SqPx and oxSSL (Fig. 8).

In general, a wide variety of lipid oxidation products such as 4-HNE [35], [42], cholesterol peroxides [44], enzymatically generated lipid hydroperoxides [46], and many others [43] have been considered endogenous signalling molecules for gene transcription. On these grounds, their roles in cell physiology and different human pathologies have been discussed. For the first time, we obtained convincing data that so far unidentified products of Sq photo-oxidation could also be regarded as lipid signalling molecules interacting with several transduction pathways in human keratinocytes. Of particular interest, Sq is present exclusively in the skin of humans, water apes, whales, otters, and other water mammals [47]. Its physiological role has been mainly discussed in terms of singlet oxygen scavenging, and thus photo-protection for the hairless human skin [2]. Our present data seem to open a new protective way of Sq action: the photo-oxidized metabolites of Sq are signalling molecules for keratinocytes, to mount adaptive/protective metabolic, proliferative, and inflammatory responses to the UV component of solar light. However, upon chronic exposure to UV or/and in the case of deregulated molecular pathways in the aged or pathological skin, photo-oxidized Sq may cause persistent inflammation and over-stimulated skin metabolism. These processes may well be further exaggerated in the oily skin, overproducing sebum Sq. Collectively, SqPx may be regarded as a novel molecular target for the prevention and treatment of UV-associated skin defects/pathologies, such as solar hyperkeratosis, acute and chronic inflammation, acne eruption, premature aging, and epidermal tumours. The exact mechanism and cellular specificity of SqPx action should be further elucidated.

## Supporting Information

Figure S1 Thin layer chromatogram of the lipid fractions of SSL, performed with elution system as described in Materials and Methods. The squalene fraction is identified, and the width of the band isolated and extracted for experiments framed in the rectangle.
(TIF)

Figure S2 GC-MS total ion chromatogram (elution conditions as described in Materials and Methods section, scan mode) of squalene isolated from irradiated SSL, confirming the purity of the extracted band. SSL sample was derivatised by N,O-bis-(trimethylsylil)-trifluoroacetamide with 1% trimethylchlorosilane.
(TIF)
