# Supplementary figures and images for: Photo-Oxidation Products of Skin Surface Squalene Mediate Metabolic and Inflammatory Responses to Solar UV in Human Keratinocytes
Source: PLoS One. 2012 Aug 30;7(8):e44472. doi: 10.1371/journal.pone.0044472 (PMC3431355; doi:10.1371/journal.pone.0044472)

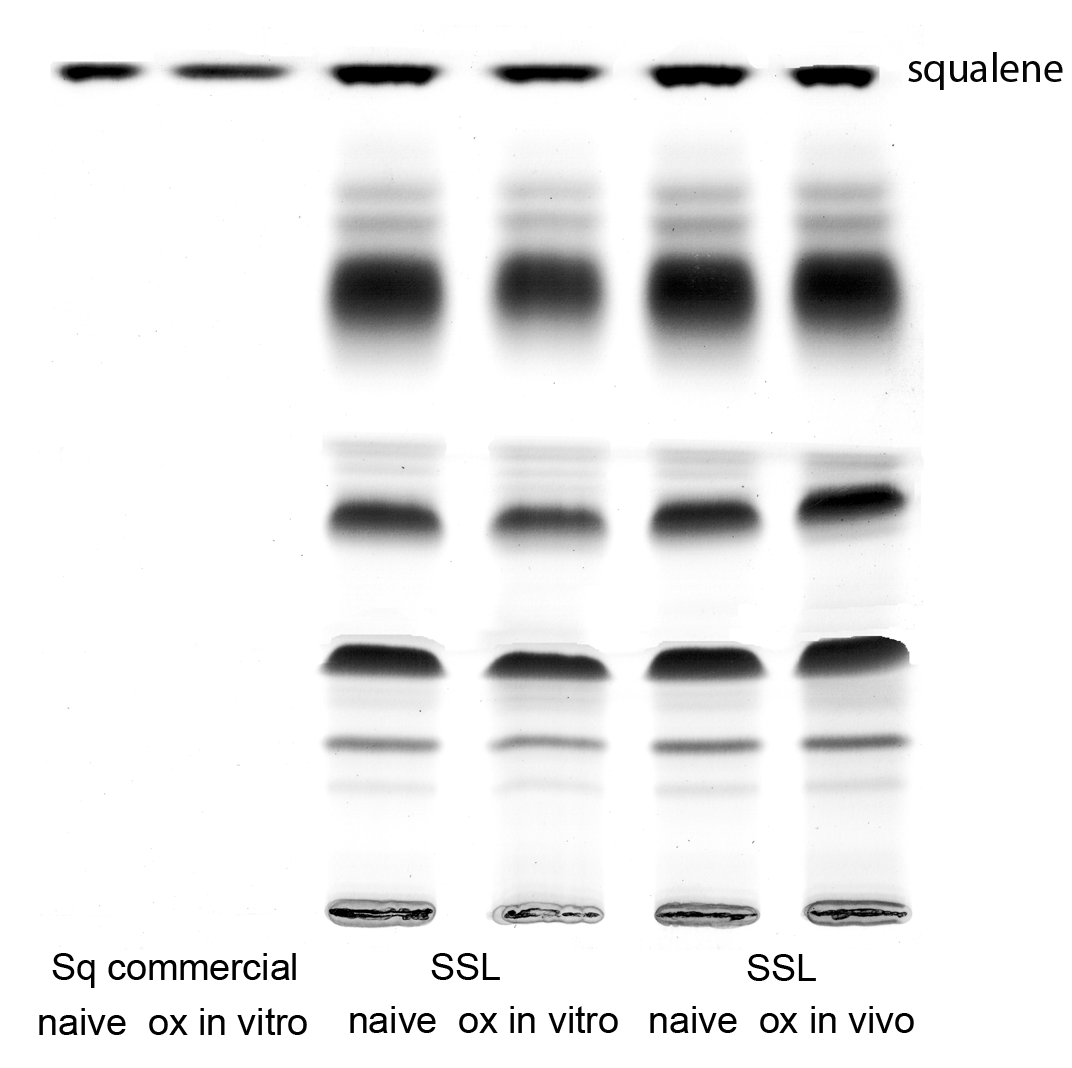

Supplement: Figure S1 — Thin layer chromatogram of the lipid fractions of SSL, performed with elution system as described in Materials and Methods. The squalene fraction is identified, and the width of the band isolated and extracted for experiments framed in the rectangle. (TIF) [file pone.0044472.s001.tif]

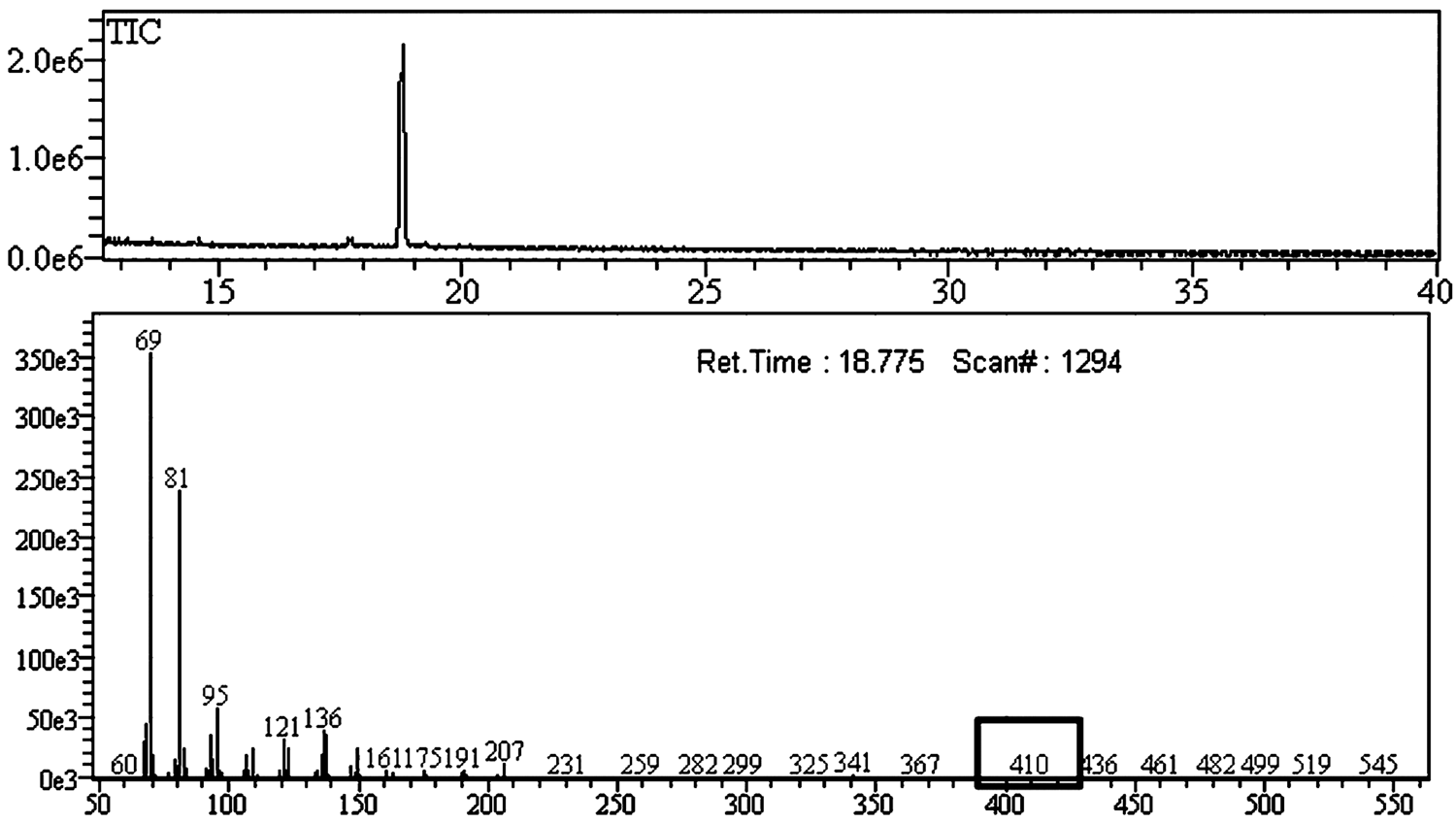

Supplement: Figure S2 — GC-MS total ion chromatogram (elution conditions as described in Materials and Methods section, scan mode) of squalene isolated from irradiated SSL, confirming the purity of the extracted band. SSL sample was derivatised by N,O-bis-(trimethylsylil)-trifluoroacetamide with 1% trimethylchlorosilane. (TIF) [file pone.0044472.s002.tif]
